# Optimal Harvesting in Stream Networks: Maximizing Biomass and Yield

**DOI:** 10.1007/s11538-026-01658-8

**Published:** 2026-05-19

**Authors:** Tung D. Nguyen, Zhisheng Shuai, Tingting Tang, Amy Veprauskas, Yixiang Wu, Ying Zhou

**Affiliations:** 1 Department of Mathematics, University of California Los Angeles, Los Angeles, CA 90024 USA; 2 Department of Mathematics, University of Central Florida, Orlando, FL 32816 USA; 3 Department of Mathematics and Statistics, San Diego State University, San Diego, CA 92182 USA; 4 Department of Mathematics, University of Louisiana at Lafayette, Lafayette, LA 70501 USA; 5 Department of Mathematical Sciences, Middle Tennessee State University, Murfreesboro, TN 37132 USA; 6 Department of Mathematical Sciences, Lafayette College, Easton, PA 18042 USA

**Keywords:** Metapopulation model, Stream network, Asymmetric movement, Harvesting, Total biomass, Sustainable yield, Network connectivity

## Abstract

In this study, we develop a metapopulation model framework to identify optimal harvesting strategies for a population in a stream network. We consider two distinct optimization objectives: maximization of total biomass and maximization of total yield, under the constraint of a fixed total harvesting effort. We examine in detail the special case of a two-patch network and fully characterize the optimal strategies for each objective. We show that when the population growth rate exceeds a critical threshold, a single harvesting strategy can simultaneously maximize both objectives. For general *n*-patch networks with homogeneous growth rates across patches, we focus on the regime of large growth rates and demonstrate that the optimal harvesting strategy selects patches according to their intraspecific competition rates and an *effective net flow* metric determined by network connectivity parameters.

## Introduction

Harvesting of renewable resources such as fish stocks provides essential benefits including food and economic livelihood. For example, the current Fisheries Economics of the United States Report by NOAA estimates that in 2023, U.S. recreational fishing and the seafood industry supported $145 and $173 billion in total sales impacts and a total of 2.1 million full-time and part-time jobs nationwide (National Marine Fisheries Service 2016). The Food and Agriculture Organization of the United Nation reports that global capture fisheries in 2022 amounted to 92.3 million tonnes and employed an estimate of 33.4 million people (FAO 2024).

Harvesting strategies are an important part of managing renewable resources. Poorly managed harvesting can cause overexploitation and resource depletion (Hilborn 2011; Agriculture Organization of the United Nations 2018). It is therefore critical to identify sustainable harvesting strategies that strike a balance between harvesting and species preservation. Mathematical models offer a useful framework for addressing this challenge. Concepts such as maximum sustainable yield in the classic bioeconomic models (Colin and Clark 2010; Gordon 1954; Schaefer 1954) have played a foundational role in learning how to optimize yield without depleting the resource. The characterization and analysis of optimal harvesting strategies have received considerable attention in the mathematical and ecological study of renewable resource management, including investigations in homogeneous environments (Brauer et al. 1975; Brauer 1976, 1979; Dunkel 1970, 2006; Swan 1975; Wan and Anderson 1983), periodic environments (Dong et al. 2007; Fan and Wang 1998; Feng et al. 2023; Liu 2022; Xiao et al. 2006; Xu et al. 2005), models with impulsive harvesting (Angelova and Dishliev 2000; Berezansky and Braverman 2004; Braverman and Mamdani 2008; Cid et al. 2014; Shuai et al. 2007; Tang et al. 2006; Zhang et al. 2003, 2006), and spatially heterogeneous settings formulated through partial differential equations (Bai and Wang 2005; Braverman and Braverman 2009; Cui et al. 2017; Ding and Lenhart 2009; Guillermo et al. 2010; Kelly et al. 2016, 2019; Korobenko and Braverman 2009; Korobenko et al. 2013; Neubert 2003; Oruganti et al. 2002) or metapopulation systems of ordinary differential equations (Auger et al. 2022; Elbetch and Moussaoui 2023; Gao and Lou 2022; González-Olivares and Huincahue-Arcos 2011; Nguyen-Ngoc et al. 2024; Pilyugin et al. 2016; Femke et al. 2025; Asep et al. 1999; Takashina and Mougi 2015; Geoffrey et al. 2000; Wu et al. 2020; Zhang et al. 2015).

While many harvesting models assume well-mixed populations, it is beneficial to consider the effect of space and dispersal in determining optimal harvesting strategies (Ding and Lenhart 2009; González-Olivares and Huincahue-Arcos 2011; Guillermo et al. 2010; Kelly et al. 2016; Asep et al. 1999; Takashina and Mougi 2015; Geoffrey et al. 2000). In Guillermo et al. (2010), the authors showed that ignoring spatial dynamics can lead to suboptimal results. In Takashina and Mougi (2015), the authors demonstrated that spatially explicit harvest models can yield lower maximum sustainable yields than their non-spatial counterparts, raising concerns of overharvesting when spatial heterogeneity is neglected. Spatially explicit models are especially important when designing marine projected areas (Steven et al. 2010; González-Olivares and Huincahue-Arcos 2011; Kelly et al. 2016; Holly et al. 2015; Neubert 2003; Holly et al. 2015) or considering the effect of spatial heterogeneity (Kelly et al. 2016) on populations. A spatially explicit model also allows us to investigate how the spatial distribution of harvesting efforts, rather than the total sum of harvesting effort, affects the population or the harvesting yield.

Besides optimizing the total yield, increasing or preserving total biomass of a fish population can also be a desirable outcome in fisheries management (Jensen 2002) because the decline or collapse of a stock is always a concern for fisheries (Lauck et al. 1998). In some ways, harvesting and preserving may not be conflictive goals: in James et al. (2001), the authors showed that both the sustainable yield and the sustainable aggregate biomass of the fish population can be increased by the creation of no-harvest marine reserves. Can we find such win-win scenarios in the context of optimization? And if we cannot optimize both the yield and the biomass, how should we direct harvesting efforts when prioritizing one over the other?

In this paper, we study a metapopulation model for a population that resides in a stream network consisting of connected habitat patches. Here, a stream network refers to a directed graph of patches with a clear notion of upstream and downstream patches and the assumption that the movement rate is higher in the downstream direction (for a more precise graph-theoretic definition, see Nguyen et al. (2023b)). Our goals are to find optimal ways to distribute harvesting efforts in the patches so as to maximize either the total biomass or the maximum sustainable yield across the patches. Our analysis builds on earlier results in Chen et al. (2022); Nguyen et al. (2023b). In particular, the sharp bounds and monotonicity results established in Chen et al. (2022) for eigenvalue problems are used to characterize population persistence, while Nguyen et al. (2023b) provides biological insights into metapopulation growth and biomass in stream networks in the absence of harvesting. In Nguyen et al. (2023b), it is found that concentrating resources (in terms of growth rates) on the most upstream patch yields the largest biomass. This insight is consistent with what we find in this paper when resources are abundant. For a homogeneous two-patch model, we find that when resources are abundant, maximum total biomass is achieved by harvesting exclusively on the downstream patch (thus preserving growth rates in the upstream patches). When resources are modest, we find that harvesting exclusively in the upstream patch (preserving growth rates downstream) maximizes biomass (Theorem 3.6; also see Theorem 3.5). This can also be interpreted, with the help of results in Nguyen et al. (2023b), that concentrating resources in the downstream patches maximizes the metapopulation growth rate, which is helpful when the growth rate is modest. Harvesting exclusively in the downstream patch also maximizes yield when growth rates are high and advection is sufficiently strong relative to the total harvest (Theorem 3.12). When these parameter conditions are not met, however, optimal strategies for maximizing the yield can vary (Figure 2). For *n*-patch networks with heterogeneous intraspecific competition rates, the two optimization objectives lead to opposite strategies: maximizing biomass favors concentrating harvesting on patches with larger competition rates (Corollary 4.3), while maximizing yield favors concentrating on patches with smaller competition rates (Corollary 4.10). Within these patches, we find that the optimal distribution of harvesting efforts for both the biomass and yield objective depends on an effective net flow metric (Definition 4.4).

The rest of the paper is organized as follows. In Section 2, the model formulation for a stream network with harvesting is presented and and the biomass and yield optimization problems are defined. Section 3 considers a special case where the network consists of only two patches. For this scenario we examine optimal harvesting strategies for both heterogeneous and homogeneous patches. Section 4 extends the results to an *n*-patch network. Finally, we discuss the findings and directions of future work in Section 5.

## Model Formulation and Optimization Problems

The classic logistic model takes the form

2.1 $$\begin{aligned} u'=ru\Big (1-\frac{u}{K}\Big ), \end{aligned}$$

where $u=u(t)$ denotes the population size (or density) at time *t*, *r* is the intrinsic growth rate, and $K>0$ is the environmental carrying capacity. This formulation presumes a nonnegative intrinsic growth rate ($r\ge 0$) and thus becomes invalid when $r<0$ as it may produce negative population size.

To allow for both positive and negative intrinsic growth, we adopt the following logistic-type modification:

2.2 $$\begin{aligned} u'=u(r-cu), \end{aligned}$$

where $r\in \mathbb {R}$ may be either positive or negative, and $c>0$ measures the strength of intraspecies competition. Model (2.2) may admit two equilibria: the trivial equilibrium $u=0$, and, when $r>0$, the positive equilibrium $u^*=\frac{r}{c}$. The stability properties are straightforward: if $r\le 0$, the trivial equilibrium $u=0$ is globally asymptotically stable in $[0, +\infty )$; if $r> 0$, then the trivial equilibrium becomes unstable and the positive equilibrium $u^*$ is globally asymptotically stable in $(0,+\infty )$. Biologically, when $r>0$, the ratio *r*/*c* can be interpreted as the environmental carrying capacity, analogous to *K* in model (2.1).

Building on the modified logistic model (2.2), we introduce the following metapopulation framework with explicit population control in a landscape of *n* patches:

2.3 $$\begin{aligned} u_i' = u_i (r_i - c_i u_i) - h_i u_i + \sum _{j=1}^n (a_{ij}u_j - a_{ji} u_i), \quad i=1,2,\ldots , n, \end{aligned}$$

where $u_i=u_i(t)$ denotes the population density in patch *i* at time *t*, $r_i$ is the intrinsic growth rate, $c_i>0$ reflects the intensity of intraspecies competition, $h_i$ represents the harvesting or stocking effort applied to patch *i*, and $a_{ij} \ge 0$ denotes the dispersal rate from patch *j* to patch *i*. Let $\bar{\boldsymbol{u}}$ denote the solution of (2.3) with no harvesting (i.e., $h_i=0$). Then we observe that

$$ \bar{u}_i'\ge \bar{u}_i (r_i - c_i \bar{u}_i) - h_i \bar{u}_i + \sum _{j=1}^n (a_{ij}\bar{u}_j - a_{ji} \bar{u}_i). $$

Thus, $\bar{\boldsymbol{u}}$ is an upper solution of (2.3), which is cooperative (see Smith (1995) for more details). So, $\bar{\boldsymbol{u}}\ge \boldsymbol{u}=(u_1, u_2, \dots , u_n)$, implying that the population decreases with harvesting effort ($h_i>0$).

We assume that the movement matrix

2.4 $$\begin{aligned} A:=\begin{pmatrix} -\sum _j a_{j1} & a_{12} & \cdots & a_{1n}\\ a_{21} & -\sum _j a_{j2} & \cdots & a_{2n}\\ \vdots & \vdots & \ddots & \vdots \\ a_{n1} & a_{n2} & \cdots & -\sum _j a_{jn} \end{pmatrix} \end{aligned}$$

is irreducible. Let *s*(*M*) denote the *spectral bound* of a matrix *M*, i.e.

$$ s(M):=\max \Big \{\textrm{Re} \lambda : \; \lambda \ \text{ is } \text{ an } \text{ eigenvalue } \text{ of } {M} \Big \}. $$

Then the metapopulation growth rate $\rho$ is defined as the spectral bound of the Jacobian matrix *J* for the linearization of (2.3) at the trivial equilibrium $(u_1,u_2,\ldots ,u_n)=(0,0,\ldots ,0)$, i.e.

2.5 $$\begin{aligned} \rho :=s(A+R), \end{aligned}$$

where $R=\textrm{diag}\{r_i-h_i\}$ and *A* is the movement matrix (2.4). As with the base model (2.2), the dynamics of the metapopulation model (2.3) are characterized by the stability of the trivial equilibrium $E_0=(0,0,\ldots , 0)$ and the positive equilibrium $E^*=(u_1^*, u_2^*, \ldots , u_n^*)$: if $\rho < 0$, then the trivial equilibrium $E_0$ is globally asymptotically stable; if $\rho >0$, then the positive equilibrium $E^*$ is globally asymptotically stable (Nguyen et al. 2023a).

Despite considerable progress in recent decades, major challenges persist in characterizing optimal harvesting strategies, largely due to the intrinsic complexity of the optimization problems and the heterogeneity inherent of the modeling framework. We illustrate these difficulties here for the case of unconstrained harvesting. When $n=1$, the maximum sustainable yield (MSY) is

$$\begin{aligned} \mathcal {Y}=\frac{r^2}{4c} \; , \end{aligned}$$

which is attained at the harvesting rate $h=\frac{r}{2}$, yielding the equilibrium population $u^*=\frac{r}{2c}$. If $h>r$, the trivial equilibrium $u=0$ is globally asymptotically stable and the population goes to extinction. Meanwhile, for general *n*, the positive equilibrium $u^*=(u_1^*, \ldots , u_n^*)$ satisfies

2.6 $$\begin{aligned} u_i^*[(r_i-h_i) - c_iu_i^*] + \sum _j (a_{ij} u_j^* - a_{ji} u_i^*) = 0, \qquad i=1,\ldots ,n. \end{aligned}$$

Summing over *i* and using the identity

$$\begin{aligned} \sum _{i,j} (a_{ij} u_j^* - a_{ji} u_i^*) = 0, \end{aligned}$$

we obtain

$$\begin{aligned} \sum _i u_i^*[(r_i-h_i)-c_iu_i^*] = 0. \end{aligned}$$

Rearranging yields an explicit expression for the total yield:

2.7 $$\begin{aligned} \mathcal {Y} = \sum _i h_i u_i^* = \sum _i r_i u_i^* - \sum _i c_i (u_i^*)^2. \end{aligned}$$

The right-hand side is maximized when $u_i^* = \frac{r_i}{2c_i}, i=1,\ldots ,n$. Substituting these values into the equilibrium equations (2.6) shows that the harvesting rates achieving maximal yield satisfy

2.8 $$\begin{aligned} h_i = \dfrac{r_i}{2} + \sum _j \Big (a_{ij}\frac{r_j/c_j}{r_i/c_i} - a_{ji}\Big ), \qquad i=1,\ldots ,n. \end{aligned}$$

These optimal harvesting strategies, which may be positive (harvesting) or negative (stocking), reflect a combination of local patch properties ($r_i$ and $c_i$) and the overall network connectivity among patches, encoded in the dispersal rates $a_{ij}$. The resulting metapopulation-level maximum sustainable yield (MSY) is given by

2.9 $$\begin{aligned} \mathcal {Y} = \sum _i \frac{r_i^2}{4c_i} \; . \end{aligned}$$

In the special case when $r_i=r$ for all *i*, the harvesting effort distribution depends solely on the sum $\sum _j \Big (a_{ij}\frac{c_i}{c_j} - a_{ji}\Big )$, which can be regarded as a “weighted" net flow into a node *i*. We show later in Section 4 that a similar quantity (Definition 4.4) also plays a vital role in maximizing the total biomass and the yield in the constrained harvesting problem.

Under the additional constrain $h_i\ge 0$ (i.e., stocking is not allowed), the MSY problem becomes considerably more complex. Moreover, in practical applications, unconstrained harvesting is not feasible; instead, harvesting effort is subject to constraints. In the remaining of the paper, we aim to provide a systematic classification of which habitat patches to control, and to what extent, in order to achieve optimal management objectives in this case. In particular, we consider the following two optimization problems

1. **Maximizing total biomass:**
  $$\begin{aligned} \max _{h_i \ge 0} \; \liminf _{t\rightarrow \infty } \sum _{i=1}^n u_i(t), \end{aligned}$$
2. **Maximizing sustainable harvesting yield:**
  $$\begin{aligned} \max _{h_i\ge 0} \liminf _{t\rightarrow \infty } \sum _{i=1}^n h_i u_i(t), \end{aligned}$$

subject to the harvesting constraint $\sum _{i=1}^n h_i = H$.

To focus the analysis, we consider two cases, when heterogeneity arises solely from the network of connections between otherwise homogeneous patches and when the patches are also heterogeneous. These cases allows us to isolate and rigorously examine the role of connectivity and patch heterogeneity in shaping both intrinsic dynamics and the effectiveness of control strategies. Our results highlight not only the critical influence of structural heterogeneity on population outcomes, but also its implications for the design of optimal interventions in fragmented or interconnected ecological systems.

## Constrained Harvesting in a Two-Patch System with Biased Movement

In this section we consider a constrained harvesting problem in a two-patch system with biased movement. We make the following assumptions:

1. We call patch 1 the *upstream patch* and patch 2 the *downstream patch* and assume that the movement rate from the upstream patch to the downstream patch is $d+q$, and the movement rate from the downstream patch to the upstream patch is *d* where $d, q>0$.
2. The total harvesting effort is fixed and there is no stocking, i.e. $h_1+h_2=H>0$ and $h_1,h_2\ge 0$.

To specify the distribution of harvesting effort, we introduce a new parameter $\theta \in [0,1]$ such that:

3.1 $$\begin{aligned} h_1=\theta H \quad \text {and} \quad h_2=(1-\theta )H. \end{aligned}$$

Combining assumptions (H1), (H2) and equation (3.1), the ODE system we consider is

3.2 $$\begin{aligned}&u_1'=u_1(r_1-\theta H -c_1u_1) -(d+q)u_1+du_2\nonumber \\&u_2'=u_2(r_2-(1-\theta )H -c_2u_2)+(d+q)u_1-du_2. \end{aligned}$$

In what follows, we utilize inequality (1.2) in Chen et al. (2022). For completeness, we include this result below.

### Lemma 3.1

(See Chen et al. (2022)) Let *A* be an essentially nonnegative irreducible matrix and let $P= \hbox {diag}\{p_i\}$ be a real diagonal matrix. Then the metapopulation growth rate $\rho$ as defined in (2.5) has the following bounds

3.3 $$\begin{aligned} \sum _{i} \alpha _i p_i \le \rho = s(A+P) \le \max \{p_i\}, \end{aligned}$$

where $(\alpha _1, \alpha _2, \ldots , \alpha _n)^T$ is the positive eigenvector of *A* corresponding to the eigenvalue 0 and satisfying $\sum _{i=1}^n \alpha _i = 1$.

Now, we present a sufficient condition for the species to persist in both patches.

### Lemma 3.2

Suppose the intrinsic growth rates $r_1$ and $r_2$ satisfy

$$ \frac{d}{2d+q}r_1+\frac{d+q}{2d+q}r_2>H\frac{d+q}{2d+q} =: r_{\text {crit}}. $$

Then there exists a unique positive equilibrium $\boldsymbol{u}^*$ which is asymptotically stable.

### Proof

From Lemma 3.1, we can obtain a lower bound for the spectral bound of the Jacobian matrix for the linearization of system (3.2) at the trivial equilibrium

$$ \rho \ge (r_1-\theta H)\phi _1 + (r_2-(1-\theta )H)\phi _2, $$

where $(\phi _1,\phi _2)$ is the normalized eigenvector of *L* corresponding to the eigenvalue 0. It is easy to check that

$$ (\phi _1,\phi _2)=\bigg (\frac{d}{2d+q},\frac{d+q}{2d+q}\bigg ). $$

Thus we have

$$\begin{aligned} \rho&\ge (r_1-\theta H)\frac{d}{2d+q} + (r_2-(1-\theta )H)\frac{d+q}{2d+q} \\&= \frac{d}{2d+q}r_1+\frac{d+q}{2d+q}r_2 - H\bigg (\theta \frac{d}{2d+q}+(1-\theta )\frac{d+q}{2d+q}\bigg )\\&\ge \frac{d}{2d+q}r_1+\frac{d+q}{2d+q}r_2-r_{\text {crit}} \end{aligned}$$

since $\theta \in [0,1]$. Therefore a sufficient condition for persistence is that

$$ \frac{d}{2d+q}r_1+\frac{d+q}{2d+q}r_2>H\frac{d+q}{2d+q} =: r_{\text {crit}}. $$

$$\square $$

Notice that this result gives a sufficient condition on the harvesting effort *H* for population persistence:

$$ H<\frac{d}{d+q}r_1+r_2. $$

For example, when $q=0$, the inequality becomes $H<r_1+r_2$, indicating that persistence can be achieved when the total harvest effort is less than the sum of the growth rates. When $d=0$, this becomes $H<r_2$, independent of the upstream growth rate.

Assuming that the species persists, let $\boldsymbol{u}^*(\theta ) = (u_1^*(\theta ),u_2^*(\theta ))$ be the unique positive equilibrium of system (3.2). For notational convenience, we omit the function arguments and the star and just write $(u_1,u_2)$ for the positive equilibrium. Thus $(u_1,u_2)$ is the solution of the following system

3.4a $$\begin{aligned}&u_1(r_1-\theta H -c_1u_1) -(d+q)u_1+du_2=0\end{aligned}$$

3.4b $$\begin{aligned}&u_2(r_2-(1-\theta )H -c_2u_2)+(d+q)u_1-du_2=0. \end{aligned}$$

### Maximizing the Total Biomass When the Patches are Heterogeneous

We first consider the optimal harvesting strategy if the goal is to maximize the (remaining) total biomass $\mathcal {M}=u_1+u_2$. We start with a lemma that provides an expression for $\mathcal {M}'(\theta )$.

#### Lemma 3.3

We have the equality

3.5 $$\begin{aligned} \mathcal {M}'(\theta )= -\frac{H(u_1+u_2)((d+q)u_1^2-du_2^2)+Hu_1^2u_2^2(c_2-c_1)}{\det (A)}, \end{aligned}$$

where

3.6 $$\begin{aligned} A=\begin{bmatrix} -(c_1u_1^2+du_2) & du_1\\ (d+q)u_2 & -(c_2u_2^2+(d+q)u_1) \end{bmatrix}. \end{aligned}$$

#### Proof

Differentiating equations (3.4a) and (3.4b) in terms of $\theta$ yields another pair of equations

3.7a $$\begin{aligned}&u_1'(r_1-\theta H-c_1u_1)-u_1(H+c_1u_1')-(d+q)u_1'+du_2'=0\end{aligned}$$

3.7b $$\begin{aligned}&u_2'(r_2-(1-\theta )H-c_2u_2) - u_2(-H+c_2u_2')+(d+q)u_1'-du_2'=0. \end{aligned}$$

By taking $u_1\times (3.7a)-u_1'\times (3.4a)$ and $u_2\times (3.7b)-u_2'\times (3.4b)$ we obtain

$$\begin{aligned}&-u_1^2(H+c_1u_1') +du_2'u_1-du_1'u_2=0\\&-u_2^2(-H+c_2u_2')+(d+q)u_1'u_2-(d+q)u_2'u_1=0. \end{aligned}$$

Rearranging terms results in

$$\begin{aligned} A\begin{pmatrix} u_1' \\ u_2'\end{pmatrix} = \begin{pmatrix} Hu_1^2\\ -Hu_2^2\end{pmatrix}, \end{aligned}$$

where the matrix *A* is given by equation (3.6). It is easy to check that $\det (A)>0$. Solving this system yields

$$\begin{aligned} \begin{pmatrix} u_1' \\ u_2'\end{pmatrix}&=\frac{-1}{\det (A)} \begin{bmatrix} c_2u_2^2+(d+q)u_1 & du_1\\ (d+q)u_2 & c_1u_1^2+du_2 \end{bmatrix}\begin{pmatrix} Hu_1^2\\ -Hu_2^2\end{pmatrix}\\&=\frac{-1}{\det (A)}\begin{pmatrix} Hu_1^2(c_2u_2^2+(d+q)u_1) - Hdu_2^2u_1\\ H(d+q)u_1^2u_2-Hu_2^2(c_1u_1^2+du_2)\end{pmatrix} \end{aligned}$$

Adding $u_1'$ and $u_2'$ and simplifying gives us equation (3.5). $\square$

From Lemma 3.3, there are two factors affecting the sign of $\mathcal {M}'$ (and thus the strategy to maximize $\mathcal {M}$).

- The first factor is the difference $(d+q)u_1^2-du_2^2$. We will show later that this difference depends on the parameters from both individual patches and the movement network.
- The second factor is the difference $c_2-c_1$, which only depends on the intraspecific competition rate of individual patches.

#### Remark 3.4

While determining the sign of $\mathcal {M}'$ is challenging in general, there are cases when one factor dominates the other, and $\mathcal {M}'(\theta )$ is guaranteed to have one sign for any $\theta \in [0,1]$.

Firstly, when $u_1$ and $u_2$ are large and of the same order of magnitude, the sign of $\mathcal {M}'(\theta )$ is determined by $c_2-c_1$. Thus the strategy to maximize $\mathcal {M}$ depends on the individual patch parameters and not on the movement rates. In particular, if $c_2>c_1$, then $\mathcal {M}$ is maximized when $\theta =0$ and, if $c_1>c_2$, then $\mathcal {M}$ is maximized when $\theta =1$. In other words, to maximize the total biomass, we concentrate the harvesting effort on the patch with the higher intraspecific competition rate. This observation is generalized to *n*-patch systems in Section 4.

On the other hand, if $c_1=c_2=c$, then the sign of $\mathcal {M}'(\theta )$ is determined by the sign of $(d+q)u_1^2-du_2^2$. We show in the next theorem that this sign depends on a “weighted difference" between the intrinsic growth rates $r_1$ and $r_2$.

#### Theorem 3.5

Consider system (3.2) and assume the persistence condition in Lemma 3.2. Assume further that $c_1=c_2=c$.

- Suppose the growth rates $r_1$ and $r_2$ satisfy
  $$\begin{aligned} \frac{r_1\sqrt{d+q}-r_2\sqrt{d}}{\sqrt{d+q}-\sqrt{d}}> 2d+q +\frac{\sqrt{d+q}}{\sqrt{d+q}-\sqrt{d}}H=:r_M. \end{aligned}$$
  Then the total biomass $\mathcal {M}(\theta )$ is decreasing with respect to the harvesting effort $\theta$ in the upstream patch. Thus $\mathcal {M}(\theta )$ is maximized at $\theta =0$, i.e. when the harvesting effort is concentrated on the downstream patch.
- Suppose the growth rates $r_1$ and $r_2$ satisfy
  $$\begin{aligned} \frac{r_1\sqrt{d+q}-r_2\sqrt{d}}{\sqrt{d+q}-\sqrt{d}}< 2d+q -\frac{\sqrt{d}}{\sqrt{d+q}-\sqrt{d}}H=:r_m. \end{aligned}$$
  Then the total biomass $\mathcal {M}(\theta )$ is increasing with respect to the harvesting effort $\theta$ in the upstream patch. Thus $\mathcal {M}(\theta )$ is maximized at $\theta =1$, i.e. when the harvesting effort is concentrated on the upstream patch.
- Suppose
  $$ r_m\le \frac{r_1\sqrt{d+q}-r_2\sqrt{d}}{\sqrt{d+q}-\sqrt{d}}\le r_M $$
  then there exists a unique $\theta ^*\in [0,1]$ such that $\mathcal {M}'(\theta ^*)=0$. Furthermore, $\theta ^*$ is a minimum of $\mathcal {M}(\theta )$ in the interval [0, 1] and $\mathcal {M}(\theta )$ is maximized at either $\theta =0$ or $\theta =1$, i.e. when the harvesting effort is concentrated on either patch.

#### Proof

Let $t:=\frac{u_1}{u_2}$. Dividing equations (3.4a) and (3.4b) by $u_1$ and $u_2$, respectively, yields

3.9a $$\begin{aligned}&r_1-\theta H -(d+q) + \frac{d}{t}=cu_1 \end{aligned}$$

3.9b $$\begin{aligned}&r_2-(1-\theta )H -d + (d+q)t = cu_2. \end{aligned}$$

By taking (3.9a)$\div$(3.9b), we obtain

$$ \frac{r_1-\theta H -(d+q) + \frac{d}{t}}{r_2-(1-\theta )H -d + (d+q)t}=t. $$

Thus *t* satisfies

$$ f(t,\theta ) : = t(r_2-(1-\theta )H -d + (d+q)t) - (r_1-\theta H -(d+q) + \frac{d}{t}) =0. $$

Consider a fixed value of $\theta$. Note that $r_2-(1-\theta )H -d + (d+q)t>0$ due to (3.9b), so $f(t,\theta )$ is an increasing function with respect to *t*.

From Lemma 3.3 and the assumption that $c_1=c_2=c$, the sign of $\mathcal {M}'(\theta )$ is the opposite of the sign of $(d+q)u_1^2-du_2^2=(d+q)u_2^2(t^2-\frac{d}{d+q})$. Thus we wish to examine the condition for $t>\sqrt{\frac{d}{d+q}}$ and $t<\sqrt{\frac{d}{d+q}}$. To this end, we use direct calculations and evaluate

3.10 $$\begin{aligned} f\bigg (\sqrt{\frac{d}{d+q}},\theta \bigg ) = \frac{\sqrt{d+q}-\sqrt{d}}{\sqrt{d+q}}\bigg (g(\theta )-\frac{r_1\sqrt{d+q}-r_2\sqrt{d}}{\sqrt{d+q}-\sqrt{d}}\bigg ), \end{aligned}$$

where $g(\theta ):=2d+q+\frac{\theta \sqrt{d+q}-(1-\theta )\sqrt{d}}{\sqrt{d+q}-\sqrt{d}}H$. It is easy to see that $g(\theta )$ is increasing in $\theta$. Furthermore, $g(0)=r_m$ and $g(1)=r_M$, where $r_m$ and $r_M$ are defined in Theorem 3.5. We now consider three cases.

**Case (a):** Suppose that $\frac{r_1\sqrt{d+q}-r_2\sqrt{d}}{\sqrt{d+q}-\sqrt{d}}>r_M$.

Since $g(\theta )$ is increasing in $\theta$, we have $\frac{r_1\sqrt{d+q}-r_2\sqrt{d}}{\sqrt{d+q}-\sqrt{d}}>r_M=g(1)\ge g(\theta )$. As a result, $f\big (\sqrt{\frac{d}{d+q}},\theta \big ) <0$, which implies $t>\sqrt{\frac{d}{d+q}}$. From Lemma 3.3 and the assumption that $c_1=c_2=c$, we conclude that $\mathcal {M}'(\theta )<0$ and $\mathcal {M}(\theta )$ is maximized at $\theta =0$.

**Case (b):** Suppose that $\frac{r_1\sqrt{d+q}-r_2\sqrt{d}}{\sqrt{d+q}-\sqrt{d}}<r_m$.

Since $g(\theta )$ is increasing in $\theta$, we have $\frac{r_1\sqrt{d+q}-r_2\sqrt{d}}{\sqrt{d+q}-\sqrt{d}}<r_m=g(0)\le g(\theta )$. As a result, $f\big (\sqrt{\frac{d}{d+q}},\theta \big ) >0$, which implies $t<\sqrt{\frac{d}{d+q}}$. From Lemma 3.3 and the assumption that $c_1=c_2=c$, we conclude that $\mathcal {M}'(\theta )>0$ and $\mathcal {M}(\theta )$ is maximized at $\theta =1$.

**Case (c):** Suppose that $r_m\le \frac{r_1\sqrt{d+q}-r_2\sqrt{d}}{\sqrt{d+q}-\sqrt{d}}\le r_M$.

In this case, there exists a unique $\theta ^*\in [0,1]$ such that $\frac{r_1\sqrt{d+q}-r_2\sqrt{d}}{\sqrt{d+q}-\sqrt{d}}=g(\theta ^*)$. Thus $f\big (\sqrt{\frac{d}{d+q}},\theta ^*\big ) =0$, which implies $t=\sqrt{\frac{d}{d+q}}$ when $\theta =\theta ^*$. From Lemma 3.3 and the assumption that $c_1=c_2=c$ we must have $\mathcal {M}'(\theta ^*)=0$.

Furthermore, for any $\theta \in [0,\theta ^*]$, we have $\frac{r_1\sqrt{d+q}-r_2\sqrt{d}}{\sqrt{d+q}-\sqrt{d}}>g(\theta )$ and thus $\mathcal {M}'(\theta )<0$. Using an identical argument, we also have $\mathcal {M}'(\theta )>0$ for any $\theta \in [\theta ^*,1]$. Thus in the interval [0, 1], the total biomass $\mathcal {M}(\theta )$ has only one local extremum $\theta ^*$, which is a minimum. This also implies that $\mathcal {M}(\theta )$ is maximized at either $\theta =0$ or $\theta =1$. $\square$

### Maximizing the Total Biomass When the Patches are Homogeneous

In this subsection, we consider the case when the two patches are homogeneous and we show that it is possible to establish a biomass-maximizing strategy for any choice of parameters. More precisely, in addition to assumptions (H1) and (H2), we make the following assumption:

(H3) The intrinsic growth rates and intraspecies competition rates of the two patches are the same, i.e. $r_1=r_2=r$ and $c_1=c_2=c$.

Under assumption (H3), system (3.2) becomes

3.11 $$\begin{aligned}&u_1'=u_1(r-\theta H -cu_1) -(d+q)u_1+du_2\nonumber \\&u_2'=u_2(r-(1-\theta )H -cu_2)+(d+q)u_1-du_2. \end{aligned}$$

The main result for this system is stated in the following theorem.

#### Theorem 3.6

Consider system (3.11).

- If $r>2d+q+\frac{H}{2}$, then $\mathcal {M}(\theta )\le \mathcal {M}(0)$ for any $\theta \in [0,1]$. In other words, the total biomass is maximized when harvesting effort is concentrated on the downstream patch.
- If $r_{\text {crit}}\le r<2d+q+\frac{H}{2}$, then $\mathcal {M}(\theta )\le \mathcal {M}(1)$ for any $\theta \in [0,1]$. In other words, the total biomass is maximized when harvesting effort is concentrated on the upstream patch.
- If $r=2d+q+\frac{H}{2}$, then $\mathcal {M}(\theta )\le \mathcal {M}(0)=\mathcal {M}(1)$ for any $\theta \in [0,1]$. In other words, the total biomass is maximized when harvesting effort is concentrated exclusively on either the upstream or the downstream patch.

To prove Theorem 3.6, we need a series of technical lemmas. We start with a corollary of Theorem 3.5. Under the assumption (H3), the weighted difference in Theorem 3.5 becomes

$$ \frac{r_1\sqrt{d+q}-r_2\sqrt{d}}{\sqrt{d+q}-\sqrt{d}} = r. $$

Thus, we have the following corollary.

#### Corollary 3.7

Consider system (3.11).

- (Large growth rate) Suppose the growth rate *r* satisfies $r>r_M$. Then the total biomass $\mathcal {M}(\theta )$ is decreasing with respect to the harvesting effort $\theta$ in the upstream patch.
- (Small growth rate) Suppose the growth rate *r* satisfies $r<r_m$ Then the total biomass $\mathcal {M}(\theta )$ is increasing with respect to the harvesting effort $\theta$ in the upstream patch.
- (Intermediate growth rate) Suppose $r_m\le r\le r_M$, then there exists a unique $\theta ^*\in [0,1]$ such that $\mathcal {M}'(\theta ^*)=0$. Furthermore, $\theta ^*$ is a minimum of $\mathcal {M}(\theta )$ in the interval [0, 1] and $\mathcal {M}(\theta )$ is maximized at either $\theta =0$ or $\theta =1$.

Focusing on the case when $r_m\le r \le r_M$, our next lemma establishes when the total biomass has the same value at $\theta =0$ and $\theta =1$.

#### Lemma 3.8

If $\mathcal {M}(0)=\mathcal {M}(1)$, we must have $r=2d+q+\frac{H}{2}$.

#### Proof

Let $(a_1,a_2)$ be the positive equilibrium when $\theta =0$, i.e. it is the solution of

3.12a $$\begin{aligned}&a_1(r-c a_1)-(d+q)a_1+da_2=0\end{aligned}$$

3.12b $$\begin{aligned}&a_2(r-H-ca_2)+(d+q)a_1-da_2=0. \end{aligned}$$

Adding equations (3.12a) and (3.12b) yields

3.13 $$\begin{aligned} r(a_1+a_2)-c(a_1^2+a_2^2)=a_2H. \end{aligned}$$

Let $(b_1,b_2)$ be the positive equilibrium when $\theta =1$, i.e. it is the solution of

3.14a $$\begin{aligned}&b_1(r-H-c b_1)-(d+q)b_1+db_2=0\end{aligned}$$

3.14b $$\begin{aligned}&b_2(r-cb_2)+(d+q)b_1-db_2=0. \end{aligned}$$

Adding equations (3.14a) and (3.14b) yields

3.15 $$\begin{aligned} r(b_1+b_2)-c(b_1^2+b_2^2)=b_1H. \end{aligned}$$

Since $\mathcal {M}(0)=\mathcal {M}(1)$, we have $a_1+a_2=b_1+b_2$. Subtracting (3.13) from (3.15) yields

3.16 $$\begin{aligned} c(b_1^2+b_2^2-a_1^2-a_2^2) = H(a_2-b_1). \end{aligned}$$

By using $a_2-b_1=b_2-a_1$ and $a_1-b_1=b_2-a_2$ in equation (3.16), we obtain

3.17 $$\begin{aligned} c(a_2-b_1)(b_2+a_1-b_1-a_2)=H(a_2-b_1) \implies 2c(a_2-b_1)(a_1-b_1) = H(a_2-b_1). \end{aligned}$$

We first argue that $a_2\ne b_1$. Assume by contradiction that $a_2=b_1$. This further implies $a_1=b_2$. Subtracting (3.12a) from (3.14b) yields

$$ (d+q)a_2-da_1 + (d+q)a_1 -da_2=0, $$

which leads to $a_1+a_2=0$, a contradiction. Thus equation (3.17) must imply $2c(a_1-b_1)=H$. Subtracting (3.14a) from (3.12a) and using $a_2-b_2=b_1-a_1$ yields

3.18 $$\begin{aligned} r(a_1-b_1)-c(a_1-b_1)(a_1+b_1) -(2d+q)(a_1-b_1)+Hb_1=0. \end{aligned}$$

By substituting $c(a_1-b_1)=H/2$ into equation (3.18), we obtain

$$ (a_1-b_1)\bigg (r-2d-q-\frac{H}{2}\bigg )=0. $$

It is easy to see that $a_1\ne b_1$ from equation (3.18), thus we must have $r=2d+q+\frac{H}{2}$. $\square$

Next, we show that the converse of Lemma 3.8 is also true.

#### Lemma 3.9

If $r=2d+q+\frac{H}{2}$, then we have $\mathcal {M}(0)=\mathcal {M}(1)$.

#### Proof

Similar to Lemma 3.8, let $(a_1,a_2)$ be the positive equilibrium when $\theta =0$, i.e. it is the solution of

3.19a $$\begin{aligned}&a_1(2d+q+\frac{H}{2}-c a_1)-(d+q)a_1+da_2=0\end{aligned}$$

3.19b $$\begin{aligned}&a_2(2d+q-\frac{H}{2}-ca_2)+(d+q)a_1-da_2=0. \end{aligned}$$

Subtracting (3.19b) from (3.19a) yields

$$ \frac{H}{2}(a_1+a_2)-c(a_1+a_2)(a_1-a_2) - q(a_1+a_2)=0 \implies a_2-a_1=\frac{q-\frac{H}{2}}{c}. $$

We substitute this into (3.19a) and solve for $a_1$ to obtain

$$ a_1=\frac{2d+\frac{H}{2}+\sqrt{(2d+\frac{H}{2})^2+4d(q-\frac{H}{2})}}{2c}, $$

which gives us

$$ \mathcal {M}(0)=a_1+a_2=2a_1+\frac{q-\frac{H}{2}}{c}=\frac{2d+q+\sqrt{(2d+\frac{H}{2})^2+4d(q-\frac{H}{2})}}{2c}. $$

Using similar calculations for the case $\theta =1$ yields

$$ \mathcal {M}(1)=\frac{2d+q+\sqrt{(2d-\frac{H}{2})^2+4d(q+\frac{H}{2})}}{2c}. $$

Finally, it is easy to check that $\mathcal {M}(0)=\mathcal {M}(1)$. $\square$

#### Lemma 3.10

When $2d+q+\frac{H}{2}<r\le r_M$, the total biomass $\mathcal {M}(\theta )$ is maximized at $\theta =0$. When $r_m\le r<2d+q+\frac{H}{2}$, the total biomass $\mathcal {M}(\theta )$ is maximized at $\theta =1$.

#### Proof

From Lemma 3.8, we have $\{(d,q,r,c,H):\mathcal {M}(0)=\mathcal {M}(1)\} = \{(d,q,r,c,H): r=2d+q+\frac{H}{2}\}$. Since $\mathcal {M}(0)-\mathcal {M}(1)$ depends continuously on the parameters *d*, *q*, *r*, *c*, *H*, it must have the same sign in each region $R_1=\{(d,q,r,c,H): r<2d+q+\frac{H}{2}\}$ and $R_2=\{(d,q,r,c,H): r>2d+q+\frac{H}{2}\}$.

It is easy to see that $\{(d,q,r,c,H): r<r_m\}\subset R_1$, so Corollary 3.7 (b) implies $\mathcal {M}(0)<\mathcal {M}(1)$ in $R_1$. Similarly, since $\{(d,q,r,c,H): r>r_M\}\subset R_2$, Corollary 3.7 (a) implies $\mathcal {M}(0)>\mathcal {M}(1)$ in $R_2$. Finally, using Corollary 3.7 (c), we can conclude that $\mathcal {M}(\theta )$ is maximized at $\theta =0$ when $2d+q+\frac{H}{2}<r\le r_M$ and maximized at $\theta =1$ when $r_m\le r<2d+q+\frac{H}{2}$. $\square$

**Fig. 1:**
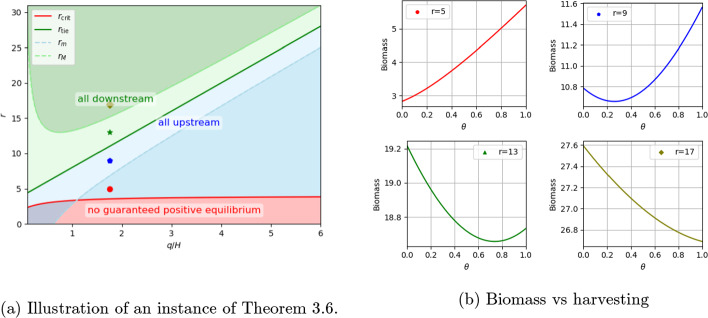
In these two figures, $r_{tie}=2d+q+\frac{H}{2}$, $d = 1$ and $H=4$, the four critical curves $r_{crit}$, $r_m,r_M,r_{tie}$ in Figure 1a separate the region to areas where the strategies for maximizing biomass are different. Four specific points are chosen with $q/H=1.75$ and different *r* values. Figure 1b shows the total biomass against the harvest parameter $\theta$ at these four points with $c=1$ and $q=7$

The proof of Theorem 3.6 now follows directly from Corollary 3.7, Lemma 3.9, and Lemma 3.10. **Figure** 1 provides an illustration of Theorem 3.6. In panel (a), the green curve $r_{\textrm{tie}}=2d+q+\tfrac{H}{2}$ partitions the $(q/H,\; r)$-plane into management regimes. Above $r_{\textrm{tie}}$ (green shading), the biomass-maximizing strategy is to harvest exclusively downstream ($\theta =0$). Biologically, advection feeds individuals into the downstream patch, where densities, thus crowding losses are highest, so removing biomass there frees capacity while preserving upstream production. Below $r_{\textrm{tie}}$ but above the persistence threshold $r_{\textrm{crit}}=\tfrac{H(d+q)}{2d+q}$ (blue shading), harvesting exclusively upstream ($\theta =1$) is optimal. Here, the intrinsic growth is relatively modest, and protecting the upstream “source” patch sustains system-wide production before individuals are flushed downstream. The red band below $r_{\textrm{crit}}$ indicates parameters with no guaranteed positive equilibrium. The four markers at $q/H=1.75$ (red, blue, green, gold) correspond to panel (b), which plots total biomass versus the harvest split $\theta$ (with $d=1$, $H=4$, $c=1$, $q=7$). Consistent with Corollary 3.7, for small $r$ (e.g., $r=5$, red), biomass increases as effort shifts upstream—protecting the source boosts overall standing stock. For large $r$ (e.g., $r=17$, gold), biomass decreases as effort moves upstream—downstream harvest removes the surplus where crowding is strongest. At intermediate $r$ (e.g., $r=13$, green), the biomass first declines and then rises with $\theta$, reflecting a transition in residence time and crowding balance across patches as the budget is reallocated. Boundaries $r_m$ and $r_M$ (dashed lines in panel (a)) demarcate these monotonicity regimes predicted by Corollary 3.7.

### Maximizing the Yield When the Patches are Homogeneous

We now turn to the optimal harvesting strategy if the goal is to maximize the yield $\mathcal {Y}(\theta )=\theta Hu_1+(1-\theta )Hu_2$. Here, we focus on the case in which the patches are homogeneous so that we are able to obtain analytical results.

Under assumption (H3), we derive an expression for $\mathcal {Y}'(\theta )$ in the following lemma.

#### Lemma 3.11

We have the equality

$$\begin{aligned} \frac{1}{H}\mathcal {Y}'(\theta )= &  \bigg [\frac{H(1-2\theta )-q}{c}+ \frac{H(1-2\theta )cu_1^2u_2^2}{\det (A)}\bigg ]- ((d+q)u_1^2-du_2^2)\\ &  \bigg [\frac{1}{cu_1u_2}+\frac{H(\theta u_1+(1-\theta )u_2)}{\det (A)}\bigg ], \end{aligned}$$

where

3.20 $$\begin{aligned} A=\begin{bmatrix} -(cu_1^2+du_2) & du_1\\ (d+q)u_2 & -(cu_2^2+(d+q)u_1) \end{bmatrix}. \end{aligned}$$

#### Proof

Differentiating the equation $\mathcal {Y}(\theta )=\theta Hu_1+(1-\theta )Hu_2$ yields

$$ \frac{1}{H}\mathcal {Y}'(\theta )= u_1-u_2 + \theta u_1' +(1-\theta )u_2'. $$

By subtracting (3.9b) from (3.9a) and using assumption (H3), we obtain

3.21 $$\begin{aligned} c(u_1-u_2)&=H(1-2\theta )-q +d\frac{u_2}{u_1} - (d+q)u_1 \implies u_1-u_2\nonumber \\&=\frac{(1-2\theta )H-q}{c} - \frac{(d+q)u_1^2-du_2^2}{cu_1u_2}. \end{aligned}$$

Using the expressions for $u_1'$ and $u_2'$ in the proof of Lemma 3.3 yields

3.22 $$\begin{aligned} \theta u_1' +(1-\theta )u_2'= - \frac{H}{\det (A)}[(2\theta -1)cu_1^2u_2^2+ (\theta u_1+(1-\theta )u_2)((d+q)u_1^2-du_2^2)]. \end{aligned}$$

Combining equations (3.21) and (3.22) and rearranging terms gives us the desired result. $\square$

Lemma 3.11 allows us to establish a sufficient condition in which $\mathcal {Y}'(\theta )<0$ and thus the yield is maximized when harvesting exclusively downstream.

#### Theorem 3.12

Suppose that $r>r_M$, where $r_M$ is defined in Theorem 3.5, and that $q\ge 2H$. Then the yield $\mathcal {Y}(\theta )$ is decreasing with respect to the harvesting effort $\theta$ in the upstream patch. Thus, the yield is maximized when harvesting is applied exclusively to the downstream patch.

#### Proof

Since $r>r_M$, we have $t=\frac{u_1}{u_2}>\sqrt{\frac{d}{d+q}}$ from the proof of Theorem 3.5 (a). Thus we have

$$ ((d+q)u_1^2-du_2^2)\bigg [\frac{1}{cu_1u_2}+\frac{H(\theta u_1+(1-\theta )u_2)}{\det (A)}\bigg ]>0 $$

since $\det (A)>0$. If $\theta \ge 1/2$, then it is clear that

$$ \frac{H(1-2\theta )-q}{c}+ \frac{H(1-2\theta )cu_1^2u_2^2}{\det (A)} <0. $$

On the other hand, if $\theta <1/2$, we first notice

$$\det (A)=(cu_1^2+du_2)(cu_2^2+(d+q)u_1)-d(d+q)u_1u_2>c^2u_1^2u_2^2. $$

Thus we have

$$ \frac{H(1-2\theta )-q}{c}+ \frac{H(1-2\theta )cu_1^2u_2^2}{\det (A)} < \frac{2H(1-2\theta )-q}{c} \le \frac{2H-q}{c}\le 0. $$

Therefore, by using Lemma 3.11, we can conclude $\mathcal {Y}'(\theta ) <0$ for any $\theta \in [0,1]$. This further implies $\mathcal {Y}(\theta )$ is maximized when $\theta =0$. $\square$

The result in Theorem 3.12 is quite intuitive. The condition $q\ge 2H$ (large advection compared to the harvesting effort) allows the biomass of the downstream patch to be large compared to the upstream patch even after harvesting. The condition $r>r_M$ (large growth rate) allows the total biomass to be maximized when harvesting exclusively downstream. Together, they naturally suggests the yield is also maximized when harvesting downstream.

When the condition in Theorem 3.12 is not satisfied, we can still use Lemma 3.11 to obtain some partial information on the distribution of harvesting effort that maximizes the yield. We state two such results in the corollary below. The proof of the corollary follows directly from Lemma 3.11.

#### Corollary 3.13

We have the following statements on the yield-maximizing effort distribution.

- (Large growth rate) Suppose that $r>r_M$, where $r_M$ is defined in Theorem 3.5. If $\theta \ge 1/2$, then the yield $\mathcal {Y}(\theta )$ is decreasing with respect to the harvesting effort $\theta$ in the upstream patch. Thus the yield is maximized at some $\theta ^*<1/2$. In other words, the yield is maximized when we harvest more on the downstream patch.
- (Small growth rate) Suppose that $r<r_m$, where $r_m$ is defined in Theorem 3.5. If $H(1-2\theta )\ge q$, or equivalently $\theta \le \frac{1}{2}-\frac{q}{2H}$, then the yield $\mathcal {Y}(\theta )$ is increasing with respect to the harvesting effort $\theta$ in the upstream patch. Thus the yield is maximized at some $\theta ^*>\frac{1}{2}-\frac{q}{2H}$.

**Fig. 2:**
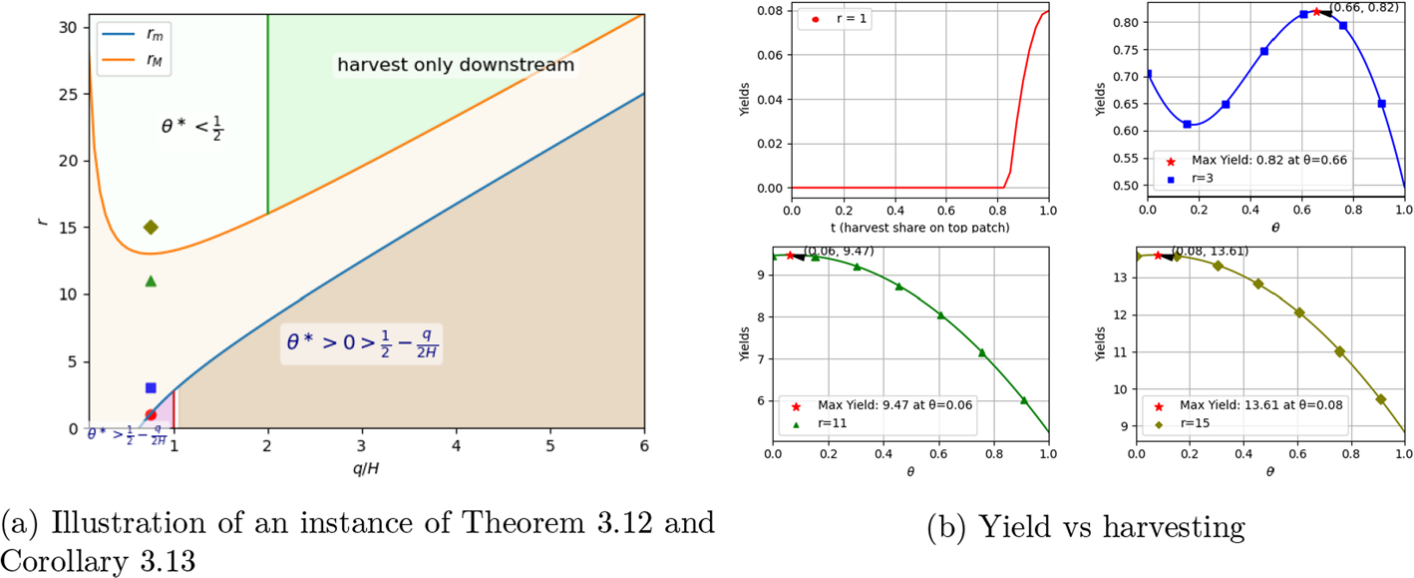
In these two figures, $d = 1$ and $H=4$, four specific points are chosen with $q/H=0.75$ and different *r* values. Figure 2b shows the yield against the harvest parameter $\theta$ at these four points with $c=1$ and $q=3$

As hinted in Corollary 3.13, the yield can follow many different patterns when the condition in Theorem 3.12 is not satisfied. **Figure** 2 presents numerical simulations illustrating these. In Figure 2a, the curves $r_m$ (blue) and $r_M$ (orange) partition the (*q*/*H*, *r*)-plane and delimit where the yield-maximizing split $\theta ^*$ (fraction of budget upstream) may occur. In the green strip, the sufficient condition $q\ge 2H$ together with large *r* ensures that the maximum sustainable yield is achieved by harvesting only downstream ($\theta ^*=0$). Below $r_m$ and $q<H$, the optimal harvesting strategy cannot be to harvest all downstream from case (*b*) in Corollary 3.13. Below $r_m$ and $q>H$ (lower tan region), the condition $\theta >\frac{1}{2}-\frac{q}{2H}$ in Corollary 3.13 is reduced to $\theta >0$, thus the corollary provides no information regarding best harvesting strategy. Figure 2b confirms these behaviors at $q/H=0.75$ (with $d=1$, $H=4$, $c=1$, and $q=3$) for four choices of *r*: for $r=1$, the yield peaks when harvesting all upstream $\theta = 1$; for $r=3$, the maximizer is interior around $\theta \approx 0.66$; for $r=11$ and $r=15$, the maximizers occur near $\theta = 0.06$ an $\theta = 0.08$, respectively. This also indicates that the optimum harvesting strategy for yield may change non-monotonously as *r* increases, further demonstrating the complexity of optimizing yield in a stream.

## Constrained Harvesting in $n-$Patch Systems

**Fig. 3:**
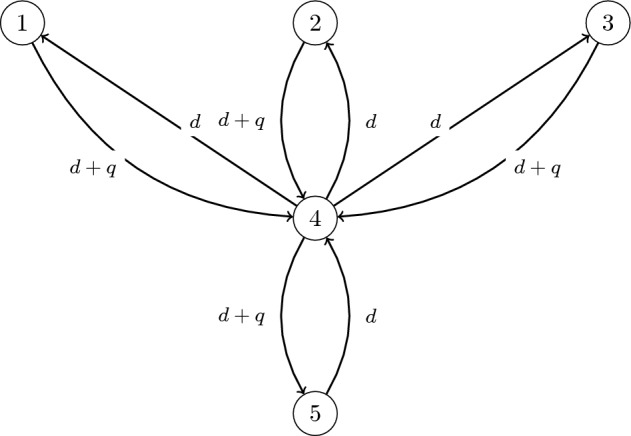
A stream network with structure 3–1–1 (three patches on top, one in the middle, one below)

In this section, we generalize some of the results in Section 3 to the *n*-patch system (2.3). In particular, we focus on the regime where the equilibrium values of $u_i$ are large. For convenience, we assume that the intrinsic growth rates are the same in each patch, i.e. $r_1=\dots =r_n=r$ and focus on the effects of the intraspecies competition rates and the movement network on the total biomass and the yield. Though the results for the two-patch system focused on a stream network, as characterized by biased movement between the patches, the results for this section apply for a more general metapopulation model as described by equation (2.3). To illustrate how these results apply to stream networks, we present two specific applications: an *n*-patch straight stream network (Examples 4.8 and 4.14) and a five-patch network (Example 4.9) whose configuration is shown in Figure 3.

### Maximizing the Total Biomass

#### Concentrating on the Patches With the Highest Intraspecies Competition Rate

Without loss of generality, that is by renumbering patches as needed, we may assume that $c_1=\dots =c_{\ell }=c>c_i$ for $i=\ell +1,\dots ,n$, i.e. the first $\ell$ patches have the strongest intraspecies competition rate.

We first show that to maximize the remaining total biomass, it is better to concentrate the harvesting effort in patch $1,\dots ,\ell$. To see this, let the harvesting effort in each patch be given as follows:

4.1 $$\begin{aligned}&h_i={\left\{ \begin{array}{ll} \beta _i(1-\theta )H \quad & \text {for} \quad i=1,\dots ,\ell \\ \alpha _i\theta H \quad & \text {for} \quad i=\ell +1,\dots ,n \end{array}\right. } \end{aligned}$$

where $\sum _{i=1}^\ell \beta _i=1$, $\sum _{i=\ell +1}^n \alpha _i=1$, and $\theta \in [0,1]$. We now show that for any choice of $\boldsymbol{\alpha }\in \mathbb {R}^{n-\ell }$ and $\boldsymbol{\beta }\in \mathbb {R}^\ell$, we must have $\lim _{r\rightarrow \infty }\mathcal {M}'<0$, where the differentiation is with respect to $\theta$.

We start with some necessary calculations. For $i=\ell +1,\dots ,n$, we have

4.2a $$\begin{aligned}&u_i(r-\alpha _i\theta H-c_iu_i) + \sum _j (a_{ij} u_j - a_{ji} u_i)=0. \end{aligned}$$

4.2b $$\begin{aligned}&u_i'(r-\alpha _i\theta H- c_iu_i)-u_i(\alpha _i H+ c_iu_i') + \sum _j (a_{ij} u_j' - a_{ji} u_i')=0, \end{aligned}$$

where the differentiation is with respect to $\theta$. For $i=1,\dots ,\ell$, we have

4.3a $$\begin{aligned}&u_i(r-\beta _i(1-\theta ) H-c_iu_i) + \sum _j (a_{ij} u_j - a_{ji} u_i)=0. \end{aligned}$$

4.3b $$\begin{aligned}&u_i'(r-\beta _i(1-\theta ) H- c_iu_i)+u_i(\beta _i H- c_iu_i') + \sum _j (a_{ij} u_j' - a_{ji} u_i')=0, \end{aligned}$$

Taking $$u_i\times (4.2b) - u_i'\times (4.2a)$$ yields

4.3c $$\begin{aligned} -u_i^2(\alpha _i H+c_iu_i')+\sum _ja_{ij}(u_j'u_i-u_ju_i')=0 \quad \text {for} \quad i=\ell +1,\dots ,n. \end{aligned}$$

Similarly, we have

4.3d $$\begin{aligned} -u_{i}^2(-\beta _iH+c_{i}u_{i}')+\sum _ja_{ij}(u_j'u_{i}-u_ju_{i}')=0 \quad \text {for} \quad i=1,\dots ,\ell . \end{aligned}$$

We apply these calculations in the following auxillary results.

##### Lemma 4.1

We have

$$ \lim _{r\rightarrow \infty } \frac{u_i}{r} = \frac{1}{c_i}. $$

Furthermore the convergence is uniform in $\boldsymbol{\alpha }\in [0, 1]^{n-\ell }$, $\boldsymbol{\beta }\in [0,1]^{\ell }$ and $\theta \in [0, 1]$.

##### Proof

Let $\kappa =1/r$ and $y_i=u_i/r$ for $i=1, \dots , n$. Then, dividing both sides of (4.2a) and (4.3a) by $r^2$, we obtain

$$\begin{aligned} \boldsymbol{f} (\kappa , \theta , \boldsymbol{\alpha }, \boldsymbol{\beta }, \boldsymbol{y}) = \textbf{0}, \end{aligned}$$

where $\boldsymbol{f}=(f_1, \dots , f_n): \mathbb {R}\times \mathbb {R}\times \mathbb {R}^{n-\ell }\times \mathbb {R}^{\ell }\times \mathbb {R}^n\rightarrow \mathbb {R}^n$ is given by

$$\begin{aligned} f_i(\kappa , \theta , \boldsymbol{\alpha }, \boldsymbol{\beta }, \boldsymbol{y})&=y_i(1-\kappa \alpha _i\theta H-c_i y_i)+\kappa \sum _j(a_{ij}y_j-a_{ji}y_i) \quad \text {for} \quad i=\ell +1,\dots , n,\\ f_{i}(\kappa , \theta , \boldsymbol{\alpha }, \boldsymbol{\beta }, \boldsymbol{y})&=y_{i}(1-\kappa (1-\beta _i)\theta H-c_{i} y_{i})+\kappa \sum _j(a_{ij}y_j-a_{ji}y_{i}) \quad \text {for} \quad i=1, \dots , \ell . \end{aligned}$$

Fix $\theta _0\in [0, 1]$, $\boldsymbol{\beta }_0=(\beta _{01},\dots ,\beta _{0\ell })\in \mathbb {R}^\ell$, and $\boldsymbol{\alpha }_0=(\alpha _{0(\ell +1)},\dots , \alpha _{0n})\in \mathbb {R}^{n-\ell }$. Let $\boldsymbol{y}_0=(y_{01},\dots , y_{0n})=(1/c_1,\dots , 1/c_n)$. It is easy to check that

$$ \boldsymbol{f}(0, \theta _0, \boldsymbol{\alpha }_0, \boldsymbol{\beta }_0, \boldsymbol{y}_0)=\boldsymbol{0}. $$

Then, we have

$$ D_{\boldsymbol{y}} \boldsymbol{f}(0, \theta _0, \boldsymbol{\alpha }_0, \boldsymbol{\beta }_0, \boldsymbol{y}_0)=-\boldsymbol{I}. $$

By the implicit function theorem, there exist $\delta >0$, open sets $U\subset \mathbb {R}$, $V\subset \mathbb {R}^{n-\ell }$ and $W\subset \mathbb {R}^\ell$ with $\theta _0\in U$, $\boldsymbol{\alpha }_0\in V$, $\boldsymbol{\beta }_0\in W$, and a unique continuous function $\boldsymbol{g}: [0, \delta ]\times U\times V\times W\rightarrow \mathbb {R}^n$ such that $\boldsymbol{y}=\boldsymbol{g}(\kappa , \theta , \boldsymbol{\alpha }, \boldsymbol{\beta })$ satisfies

$$ \boldsymbol{f}(\kappa , \theta , \boldsymbol{\alpha }, \boldsymbol{\beta }, \boldsymbol{g}(\kappa ,\theta ,\boldsymbol{\alpha }, \boldsymbol{\beta }))=\boldsymbol{0}, \ \ (\kappa , \theta , \boldsymbol{\alpha },\boldsymbol{\beta })\in [0, \delta ]\times U\times V\times W, $$

and

$$ \boldsymbol{y}_0=\boldsymbol{g}(0, \theta _0, \boldsymbol{\alpha }_0, \boldsymbol{\beta }_0). $$

By the compactness of [0, 1], $[0, 1]^{n-\ell }$ and $[0, 1]^{\ell }$, there exists $\delta _m>0$ such that all the solutions of $\boldsymbol{f}(\kappa , \theta , \boldsymbol{\alpha }, \boldsymbol{\beta }, \boldsymbol{y})=\boldsymbol{0}$ for $\kappa \in [0, \delta _m]$, $\theta \in [0, 1]$, $\boldsymbol{\alpha }\in [0, 1]^{n-\ell }$ and $\boldsymbol{\beta }\in [0, 1]^\ell$ satisfy $\boldsymbol{y}=\boldsymbol{g}(\kappa , \theta , \boldsymbol{\alpha },\boldsymbol{\beta })$. Moreover, $\boldsymbol{y}_0=\boldsymbol{g}(0, \theta , \boldsymbol{\alpha },\boldsymbol{\beta })$ for $\theta \in [0, 1]$, $\boldsymbol{\alpha }\in [0, 1]^{n-\ell }$ and $\boldsymbol{\beta }\in [0, 1]^{\ell }$. Hence, we may choose $\delta _m$ small such that $\boldsymbol{g}$ is positive.

Since *g* is continuous on the compact set $[0, \delta _m]\times [0, 1]\times \mathbb {R}^{n-\ell }\times \mathbb {R}^\ell$, it is uniformly continuous. This implies $\lim _{\kappa \rightarrow 0^+} \boldsymbol{g}(\kappa , \theta , \boldsymbol{\alpha },\boldsymbol{\beta })=\boldsymbol{g}(0, \theta , \boldsymbol{\alpha },\boldsymbol{\beta })=\boldsymbol{y}_0$ uniformly for $\theta \in [0, 1]$, $\boldsymbol{\alpha }\in [0, 1]^{n-\ell }$ and $\boldsymbol{\beta }\in [0,1]^ \ell$. By this and the uniqueness of the positive solution of (2.3), we obtain the desired result. $\square$

##### Proposition 4.2

We have

$$\begin{aligned}&\lim _{r\rightarrow \infty } u_i' = {\left\{ \begin{array}{ll} \frac{-\alpha _iH}{c_i} \quad & \text {for}\quad i=\ell +1,\dots ,n\\ \frac{\beta _i H}{c_i} \quad & \text {for}\quad i=1,\dots ,\ell . \end{array}\right. } \end{aligned}$$

Furthermore the convergence is uniform in $\boldsymbol{\alpha }\in [0, 1]^{n-\ell }$, $\boldsymbol{\beta }\in [0,1]^{\ell }$ and $\theta \in [0, 1]$.

##### Proof

By (4.3c)-(4.3d), $\boldsymbol{u}'=(u_1', \dots , u_n')^T$ satisfies $B\boldsymbol{u}'=\boldsymbol{R}$ with $R=(R_1, \dots , R_n)^T$, $R_i=\alpha _i H$ for $i=\ell +1,\dots ,n$ and $R_{i}=-\beta _i H$ for $i=1,\dots ,\ell$, and

$$\begin{aligned} B_{ij}= \left\{ \begin{array}{lll} \frac{a_{ij}}{u_i}, \ \  & {i\ne j},\\ -\sum _{j} a_{ij}\frac{u_j}{u_i^2}-c_i, & i=j. \end{array} \right. \end{aligned}$$

By Lemma 4.1, we know $B\rightarrow -\text {diag}(c_1,\dots , c_n)$ as $r\rightarrow \infty$ uniformly in $\boldsymbol{\alpha }\in [0, 1]^{n-\ell }$, $\boldsymbol{\beta }\in [0,1]^{\ell }$ and $\theta \in [0, 1]$. The desired result follows from this observation. $\square$

Using the assumption that $c_1=\dots =c_\ell =c>c_i$ for all $i=\ell +1,\dots ,n$, and the fact that $\sum _{i=1}^\ell \beta _i=1$ and $\sum _{i=\ell +1}^n\alpha _i=1$, we have the following corollary.

##### Corollary 4.3

We have

$$ \lim _{r\rightarrow \infty }\frac{1}{H}\mathcal {M}'= \frac{1}{c}-\sum _{i=\ell +1}^n\frac{\alpha _i}{c_i}< \frac{1}{c}-\frac{1}{\max _{j\in [\ell +1,n]}c_j} <0. $$

By Corollary 4.3, the total biomass $\mathcal {M}$ is decreasing in $\theta$ when *r* is large. This implies the best strategy to maximize $\mathcal {M}$ is to concentrate the harvesting effort on patches $1,\dots ,\ell$, i.e. the patches with the highest intraspecies competition rate.

#### Harvesting Effort Distribution Among the Patches With the Highest Intraspecies Competition Rate

The remaining question is to determine which harvesting effort distribution among the patches with the highest intraspecies competition rate yields the maximum biomass. We show that the answer depends on the structure of the movement network. In particular, we define a quantity for each node as follows.

##### Definition 4.4

For each node *i*, the *effective net flow* $I_i$ is defined by

$$ I_i= \sum _j\bigg (\frac{a_{ij}}{c_j}-\frac{a_{ji}}{c_i} \bigg ). $$

We show that to maximize the total biomass, among the patches $i=1,\dots ,\ell$, we concentrate all harvesting effort on the patch with the highest effective net flow. Without loss of generality, assume $I_1>I_i$ for all $i=2,\dots ,\ell$. To this end, let

4.4 $$\begin{aligned} h_1=(1-\delta )H, \quad h_i=\gamma _i\delta H \quad \text {for} \ i=2,\dots ,\ell , \quad \text {and}\quad h_i=0 \quad \text {for}\ i=\ell +1, \dots , n. \end{aligned}$$

where $$\sum _{i=2}^\ell \gamma _i=1$$ and $$\delta \in [0,1]$$.

The following results, Lemma 4.5 and Proposition 4.6, are similar to Lemma 4.1 and Proposition 4.2 and thus their proofs are omitted.

##### Lemma 4.5

We have

$$ \lim _{r\rightarrow \infty } \frac{u_i}{r} = \frac{1}{c_i}. $$

Furthermore the convergence is uniform in $\boldsymbol{\gamma }\in [0, 1]^{\ell -1}$ and $\delta \in [0, 1]$.

##### Proposition 4.6

We have

$$\begin{aligned}&\lim _{r\rightarrow \infty } u_i' = {\left\{ \begin{array}{ll} \frac{H}{c_i} =\frac{H}{c} \quad & \text {for}\quad i=1\\ \frac{-\gamma _i H}{c_i}=\frac{-\gamma _i H}{c} \quad & \text {for}\quad i=2,\dots ,\ell \\ 0 \quad & \text {for}\quad i=\ell +1,\dots ,n. \end{array}\right. } \end{aligned}$$

Furthermore the convergence is uniform in $\boldsymbol{\gamma }\in [0, 1]^{\ell -1}$ and $\delta \in [0, 1]$.

Based on Proposition 4.6 and the assumption that $c_1=\dots =c_\ell$, we have $\lim _{r\rightarrow \infty }\mathcal {M}' = 0$. To determine the sign of $\mathcal {M}'$ for large *r*, we compute the limit of $r\mathcal {M}'$ instead.

##### Proposition 4.7

We have

$$\begin{aligned} \lim _{r\rightarrow \infty } r\mathcal {M}' = H(-I_{1}+\sum _{i=2}^\ell \gamma _i I_i). \end{aligned}$$

Furthermore the convergence is uniform in $\boldsymbol{\gamma }\in [0, 1]^{\ell -1}$ and $\delta \in [0, 1]$. As a consequence, we have

$$ \lim _{r\rightarrow \infty } \frac{r}{H}\mathcal {M}'=-I_{1}+\sum _{i=2}^\ell \gamma _i I_i< - I_1+\max _{i\in [2,\ell ]}I_i<0. $$

##### Proof

Using the same calculations that lead to (4.3c) and (4.3d), we can obtain the following equations

$$\begin{aligned}&-u_i^2(-H+c_iu_i')+\sum _j a_{ij}(u_j'u_i-u_ju_i')=0 \quad \text {for} \quad i=1\\&-u_i^2(\gamma _iH+c_iu_i')+\sum _j a_{ij}(u_j'u_i-u_ju_i')=0 \quad \text {for} \quad i=2,\dots ,\ell \\&-u_i^2c_iu_i'+\sum _j a_{ij}(u_j'u_i-u_ju_i')=0 \quad \text {for} \quad i=\ell +1,\dots ,n. \end{aligned}$$

Multiplying each equation by $\frac{r}{c_iu_i^2}$ and rearranging terms yields

$$\begin{aligned}&ru_i' - \frac{Hr}{c_i}=\frac{r}{c_iu_i} \sum _ja_{ij}(u_j'-\frac{u_j}{u_i}u_i')\quad \text {for} \quad i=1\\&ru_i' + \frac{\gamma _iHr}{c_i}=\frac{r}{c_iu_i} \sum _ja_{ij}(u_j'-\frac{u_j}{u_i}u_i') \quad \text {for} \quad i=2,\dots ,\ell \\&ru_i'=\frac{r}{c_iu_i} \sum _ja_{ij}(u_j'-\frac{u_j}{u_i}u_i') \quad \text {for} \quad i=\ell +1,\dots ,n. \end{aligned}$$

Adding all equations above and using the assumption that $c_1=\dots =c_\ell =c$ and $\sum _{i=2}^\ell \gamma _i=1$ yields

$$\begin{aligned} r\mathcal {M}'= \sum _i \frac{r}{c_iu_i} u_i'\sum _j \left( a_{ji} - a_{ij} \frac{u_j}{u_i}\right) . \end{aligned}$$

Finally, taking the limit $r\rightarrow \infty$ and using Lemma 4.5 and Proposition 4.6 gives us the desired result. $\square$

From Proposition 4.7, we conclude that when *r* is large, the total biomass is maximized when $\delta =0$, i.e. among the patches with the highest $c_i$, we concentrate all harvesting effort on the patch with the highest effective net flow.

##### Example 4.8

Consider an *n*-patch straight stream network with homogeneous patches (i.e., $r_i=r$ and $c_i=c$ for all $i=1,\dots ,n$). Assume further that the drift and diffusion are also homogeneous, i.e., the movement rate from an upstream patch to a downstream patch is $d+q$, and the movement rate from a downstream patch to an upstream patch is *d*. Then it is easy to check that the most downstream patch has the maximum effective net flow. Thus, when *r* is large, we can maximize the (remaining) total biomass by harvesting the most downstream patch exclusively.

##### Example 4.9

**Fig. 4:**
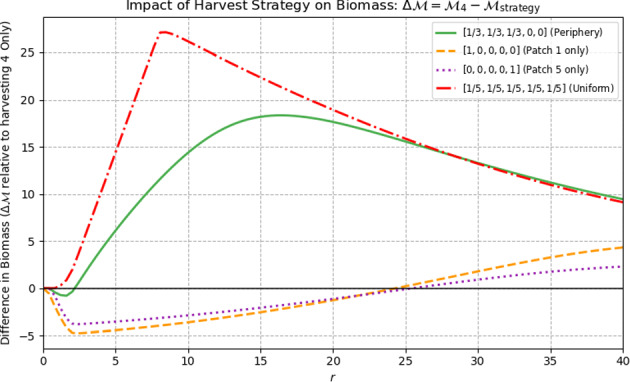
For a stream network with structure 3–1–1 (three patches on top, one in the middle, one below), the figure compares stabilized total biomass across five harvesting strategies as the intrinsic growth rate *r* increases

The most downstream patch is not always the patch with the maximum effective net flow even if we assume homogeneous patches and homogeneous drift and diffusion. In this example, we consider the 5-patch stream network shown in Figure 3. It is straightforward to verify that patch 4 has the maximum effective net flow. We illustrate this result numerically. In Figure 4, we plot $\Delta \mathcal {M}^*= \mathcal {M}^*_{4} - \mathcal {M}^*_{\text {strategy}}$, where $\mathcal {M}^*_{4}$ is the resulting biomass when only patch 4 is harvested. Thus, values above 0 indicate that harvesting only the middle patch (patch 4) yields more biomass than the alternative strategy, while values below 0 indicate the alternative leaves more biomass. The solid green curve corresponds to harvesting evenly across the three top (periphery) patches $[\tfrac{1}{3},\tfrac{1}{3},\tfrac{1}{3},0,0]$; the orange dashed curve is harvesting only the upstream-left patch [1, 0, 0, 0, 0]; the purple dotted curve is harvesting only the bottom patch [0, 0, 0, 0, 1]; the red dash–dotted curve is uniform harvesting over all five patches $[\tfrac{1}{5},\tfrac{1}{5},\tfrac{1}{5},\tfrac{1}{5},\tfrac{1}{5}]$; and the black dash–dotted line at 0 is the reference. For small *r*, the patch 1-only and patch 5-only strategies (orange, purple) yield slightly *more* biomass than patch 4 (negative $\Delta \mathcal {M}^*$), but as *r* increases they cross the reference near $r\approx 25$ and thereafter leave less biomass than harvesting only patch 4.

### Maximizing the Yield

#### Concentrating on the Patches With the Smallest Intraspecies Competition Rate

Without loss of generality, we may assume that $c_1=\dots =c_{\ell }=c<c_i$ for $i=\ell +1,\dots ,n$, i.e. the first $\ell$ patches have the smallest intraspecies competition rate.

We first show that to maximize the yield, it is better to concentrate the harvesting effort in patch $1,\dots ,\ell$. To see this, let the harvesting effort in each patch be given as follows:

4.5 $$\begin{aligned}&h_i={\left\{ \begin{array}{ll} \beta _i(1-\theta )H \quad & \text {for} \quad i=1,\dots ,\ell \\ \alpha _i\theta H \quad & \text {for} \quad i=\ell +1,\dots ,n \end{array}\right. } \end{aligned}$$

where $\sum _{i=1}^\ell \beta _i=1$, $\sum _{i=\ell +1}^n \alpha _i=1$, and $\theta \in [0,1]$. We now show that for any choice of $\boldsymbol{\alpha }\in \mathbb {R}^{n-\ell }$ and $\boldsymbol{\beta }\in \mathbb {R}^\ell$, we must have $\lim _{r\rightarrow \infty }\mathcal {Y}'<0$, where the differentiation is with respect to $\theta$. Using Lemma 4.1 and Proposition 4.2 we obtain the following corollary.

##### Corollary 4.10

We have

$$ \lim _{r\rightarrow \infty }\frac{\mathcal {Y}'}{rH} =-\frac{1}{c}+\sum _{i=\ell +1}^n \frac{\alpha _i}{c_i}<-\frac{1}{c}+\frac{1}{\min _{j\in [\ell +1,n]}c_j} <0. $$

Furthermore the convergence is uniform in $\boldsymbol{\alpha }\in [0, 1]^{n-\ell }$, $\boldsymbol{\beta }\in [0,1]^{\ell }$, and $\theta \in [0, 1]$.

##### Proof

We have

$$ \mathcal {Y}=\sum _{i=1}^\ell \beta _i(1-\theta )Hu_i+\sum _{i=\ell +1}^n \alpha _i\theta H u_i. $$

Thus

$$ \frac{\mathcal {Y}'}{rH}=\frac{1}{r}\sum _{i=1}^\ell (u_i'\beta _i(1-\theta )-u_i\beta _i)+\sum _{i=\ell +1}^n (u_i'\alpha _i \theta + u_i\alpha _i). $$

Using Lemma 4.1, Proposition 4.2, and the assumption that $c_1=\dots =c_\ell =c<c_i$ for $i=\ell +1,\dots ,n$, we have

$$ \lim _{r\rightarrow \infty } \frac{\mathcal {Y}'}{rH} = -\sum _{i=1}^\ell \frac{\beta _i}{c_i}+\sum _{i=\ell +1}^n\frac{\alpha _i}{c_i} =-\frac{1}{c}+\sum _{i=\ell +1}^n \frac{\alpha _i}{c_i}<-\frac{1}{c}+\frac{1}{\min _{j\in [\ell +1,n]}c_j} <0. $$

$$\square $$

By Corollary 4.10, the yield $\mathcal {Y}$ is decreasing in $\theta$ when *r* is large. This implies the best strategy to maximize $\mathcal {Y}$ is to concentrate the harvesting effort on patches $1,\dots ,\ell$, i.e. the patches with the smallest intraspecies competition rate.

#### Harvesting Effort Distribution Among the Patches With the Smallest Intraspecies Competition Rate

We now determine which harvesting effort distribution among the patches with the smallest intraspecies competition rate yields the maximum sustainable yield. We show that the answer depends on the structure of the movement network. In particular, we show that, under some technical conditions, we can maximize the yield by concentrating the harvesting effort on the patch with the highest effective net flow. Without loss of generality, assume $I_1>I_i$ for all $i=2,\dots ,\ell$. To this end, let

4.6 $$\begin{aligned} h_1=(1-\delta )H, \quad h_i=\gamma _i\delta H \quad \text {for} \ i=2,\dots ,\ell , \quad \text {and} \quad h_i=0\quad \text {for}\ i=\ell +1, \dots , n, \end{aligned}$$

where $$\sum _{i=2}^\ell \gamma _i=1$$ and $$\delta \in [0,1]$$.

It is easy to verify that Lemma 4.5 and Proposition 4.6 still hold.

##### Proposition 4.11

We have

$$ \mathcal {Y}'=\frac{2H}{c}\left( 1-\delta -\delta \sum _{i=2}^\ell \gamma _i^2\right) -I_1+\sum _{i=2}^\ell \gamma _iI_i. $$

##### Proof

We have

$$ \mathcal {Y}=(1-\delta )Hu_1 +\sum _{i=2}^\ell \gamma _i\delta Hu_i. $$

Thus

4.7 $$\begin{aligned} \frac{\mathcal {Y}'}{H}=(1-\delta )u_1'+\delta \sum _{i=2}^\ell \gamma _iu_i'-u_1+\sum _{i=2}^\ell \gamma _iu_i = (1-\delta )u_1'+\delta \sum _{i=2}^\ell \gamma _iu_i' + \sum _{i=2}^\ell \gamma _i(u_i-u_1). \end{aligned}$$

By dividing each equation at equilibrium by the corresponding $u_i$, we obtain

4.8 $$\begin{aligned}&r-(1-\delta )H-cu_1+\frac{1}{u_1}\sum _j (a_{1j}u_j-a_{j1}u_1)=0\end{aligned}$$

4.9 $$\begin{aligned}&r-\gamma _i\delta H-cu_i+\frac{1}{u_i}\sum _j (a_{ij}u_j-a_{ji}u_i)=0, \ i=2, \dots , \ell . \end{aligned}$$

Taking the difference (4.8) − (4.9) yields

4.10 $$\begin{aligned} c(u_i-u_1)=(1-\delta -\gamma _i\delta )H+\frac{1}{u_i}\sum _j (a_{ij}u_j-a_{ji}u_i) - \frac{1}{u_1}\sum _j (a_{1j}u_j-a_{j1}u_1), \end{aligned}$$

for $i=2,\dots , \ell$. Taking the limit $r\rightarrow \infty$ and using Lemma 4.5 yields

4.11 $$\begin{aligned} \lim _{r\rightarrow \infty } (u_i-u_1)=\frac{(1-\delta -\gamma _i\delta )H}{c}+I_i-I_1. \end{aligned}$$

Combining equations (4.7), (4.11), and the results in Proposition 4.6, we have

$$ \lim _{r\rightarrow \infty } \frac{\mathcal {Y}'}{H}= \frac{2H}{c}(1-\delta -\delta \sum _{i=2}^\ell \gamma _i^2) -I_1+\sum _{i=2}^\ell \gamma _iI_i. $$

$$\square $$

##### Remark 4.12

Proposition 4.11 allows us to obtain generalizations of the results in Section 3.3. Firstly, if the movement rates are large enough compare to the total harvesting effort *H*, then we can maximize the yield by concentrating the harvesting effort on the patch with the maximum effective net flow (among the patches with the smallest instraspecies competition rates).

The corollary below follows directly from Proposition 4.11.

##### Corollary 4.13

Suppose $$I_1-\max _{i\in [2,\ell ]} I_i > \frac{2H}{c}$$, then

$$ \lim _{r\rightarrow \infty } \frac{\mathcal {Y}'}{H}< \frac{2H}{c} - (I_1-\max _{i\in [2,\ell ]} I_i ) <0. $$

Thus $\mathcal {Y}(\delta )$ is maximized when $\delta =0$, i.e. all harvesting effort is concentrated in patch 1, which is the patch with the highest effective net flow.

##### Example 4.14

For *n*-patch straight stream network with homogeneous patches and homogeneous flow, the effective net flow of patch 1 is $-q/c$, the effective net flow of patch *n* is *q*/*c*, and the rest of the patches have 0 effective net flow. So the condition in Corollary 4.13 becomes $q>2H$, which is similar to the condition in Theorem 3.12.

Even when the condition in Corollary 4.13 is not fulfilled, we can still obtain some partial results similar to Corollary 3.13. It is easy to see that Corollary 3.13 is a special case of Corollary 4.15 when $n=2$.

##### Corollary 4.15

Suppose that $\delta >\frac{n-1}{n}$, then $\lim _{r\rightarrow \infty } \frac{\mathcal {Y}'}{H}<0$. Thus the yield $\mathcal {Y}$ is maximized at some $\delta ^*<\frac{n-1}{n}$. In other words, to maximize the yield the harvesting effort in patch 1 (the patch with the highest effective net flow) must exceed *H*/*n*.

##### Proof

We have the inequality

$$ \sum _{i=2}^\ell \gamma _i^2 \ge \frac{(\sum _{i=2}^\ell \gamma _i)^2}{n-1} = \frac{1}{n-1}. $$

Thus from Proposition 4.11, if $\delta >(n-1)/n$, we have

$$\begin{aligned} \lim _{r\rightarrow \infty } \frac{\mathcal {Y}'}{H}= &  \frac{2H}{c}\left( 1-\delta -\delta \sum _{i=2}^\ell \gamma _i^2\right) -I_1+\sum _{i=2}^\ell \gamma _iI_i \\< &  \frac{2H}{c}\left( 1-\delta \frac{n}{n-1}\right) -I_1+\sum _{i=2}^\ell \gamma _iI_i<\frac{2H}{c}\left( 1-\delta \frac{n}{n-1}\right) <0. \end{aligned}$$

$$\square $$

## Discussion

We studied how to allocate a fixed harvesting budget across patches in a stream network to optimize two objectives: maximizing the total biomass and maximizing sustainable yield in the presence of biased movement. Overall, the findings suggest that for a stream network with spatial heterogeneity, the patch parameters determine the optimal strategy whereas for homogeneous stream network, the network structure determines the optimal strategies.

For the homogeneous two-patch model, we obtained an analytical classification for maximizing biomass. Our findings show that when resources are abundant, harvesting exclusively on the downstream patch gives the largest total biomass, this result agrees with Nguyen et al. (2023b) where concentrating resources on the upstream patch yields the largest biomass. On the other hand, when resources are modest, harvesting only upstream results in maximum biomass. Given that Nguyen et al. (2023b) shows concentrating resources downstream promotes the growth rate (that is favoring downstream, similar to harvest exclusively upstream here), this could indicate the importance of promoting the growth rate when the resources are modest or scarce.

In addition, our results indicate a similar strategy for maximizing yield for the two-patch stream network, but with much more complexity. We derived parameter conditions guaranteeing that harvesting exclusively downstream maximizes yield, particularly in a high-growth regime with sufficiently strong advection relative to the harvest budget. Outside this regime, the yield-maximizing strategy can occur upstream, downstream, or at an interior split, consistent with the numerical examples and highlighting the stronger sensitivity of yield to the spatial distribution of equilibrium densities. It remains an interesting question to explore strategies for maximizing yield further for intermediate growth rates.

For *n*-patch networks, our analysis focuses on the case when *r* is large, which allows a tractable separation between (a) which subset of patches should receive harvest and (b) how to allocate harvest within that subset. With heterogeneous intraspecific competition rates, we found the opposite strategy depends on the objective: maximizing biomass favors concentrating harvest on patches with larger competition rates, while maximizing yield favors concentrating harvest on patches with smaller competition rates. However, for homogeneous stream network, the structure enters through an effective net-flow metric that identifies where concentrating harvest is most effective; in particular, the decisive patch need not be the most downstream node when connectivity creates strong transport asymmetries and the same strategy apply to maximize both biomass and yield.

While this paper focuses on two optimization problems of maximizing total biomass and yield under harvesting constraints, other optimization questions naturally arise in the context of heterogeneous population control or preservation, as well as in infectious disease management (e.g., see D’Agata et al. (2012)) and tumor/cancer control (e.g., see Hillen and Painter (2009)). We plan to investigate some of these questions in future work:

- **Maximizing total population under patch-specific constraints.** Distribute a fixed control budget $H>0$ across patches with patch specific constraints to maximize the long-term total population:
  $$\begin{aligned} \max _{\underline{h}_i^*\le h_i \le \bar{h}_i^*} \; \limsup _{t\rightarrow \infty } \sum _{i=1}^n u_i(t) \end{aligned}$$
  subject to $$\sum _{i=1}^n h_i \le H$$.
- **Minimum total control effort for extinction.** Find the least total control required across all patches to drive the metapopulation to extinction. That is,
  $$\begin{aligned} \min _{h_i\ge 0} \; \sum _{i=1}^n h_i \end{aligned}$$
  subject to $u_i(t) \rightarrow 0$ as $t\rightarrow \infty$, $i=1,2,\ldots , n$.
- **Optimal patch selection.** Minimize the number of patches under active control while still achieving extinction:
  $$\begin{aligned} \min \#\{i: h>0\} \end{aligned}$$
  subject to $u_i(t)\rightarrow 0$ as $t\rightarrow \infty$, $i=1,2,\ldots , n$.
- **Targeted control under budget constraints.** Distribute a fixed control budget $H>0$ across patches to minimize the long-term total population:
  $$\begin{aligned} \min _{h_i \ge 0} \; \limsup _{t\rightarrow \infty } \sum _{i=1}^n u_i(t) \end{aligned}$$
  subject to $$\sum _{i=1}^n h_i \le H$$.

We note that some cases of the last question can easily be resolved following the derivative of $\mathcal {M}$ and $\mathcal {Y}$, giving us mirroring results to Theorems 3.6 and 3.12, but special cases such as *c*) in Theorem 3.6 warrant further investigation, as minimization occurs when harvesting effort is split between up and down stream.

## Data Availability

There was no data generated during the study.
